# Unleashing the Potential of Bacterial Isolates from Apple Tree Rhizosphere for Biocontrol of *Monilinia laxa*: A Promising Approach for Combatting Brown Rot Disease

**DOI:** 10.3390/jof9080828

**Published:** 2023-08-05

**Authors:** Fatemeh Derikvand, Eidi Bazgir, Moussa El Jarroudi, Mostafa Darvishnia, Hossein Mirzaei Najafgholi, Salah-Eddine Laasli, Rachid Lahlali

**Affiliations:** 1 Plant Pathology, Faculty of Agriculture, Lorestan University, Lorestan, Khorramabad 68151-44316, Iran; fatemehderikvand93@gmail.com (F.D.); mdarvishnia44@yahoo.com (M.D.); mirzaeih89@gmail.com (H.M.N.); 2 Water, Environment and Development Unit, Department of Environmental Sciences and Management, UR SPHERES Research Unit, University of Liège, 6700 Arlon, Belgium; meljarroudi@uliege.be; 3 Phytopathology Unit, Department of Plant Protection, Ecole Nationale d’Agriculture de Meknès, Km10, Rte Haj Kaddour, BP S/40, Meknès 50001, Morocco; laaslisalaheddine@gmail.com; 4 Plant Pathology Laboratory, AgroBioSciences, College of Sustainable Agriculture and Environmental Sciences, Mohammed VI Polytechnic University, Lot 660, Hay Moulay Rachid, Ben Guerir 43150, Morocco

**Keywords:** apple, biocontrol, brown rot, chitinase, protease, *Monilinia laxa*, rhizosphere

## Abstract

*Monilinia laxa*, a notorious fungal pathogen responsible for the devastating brown rot disease afflicting apples, wreaks havoc in both orchards and storage facilities, precipitating substantial economic losses. Currently, chemical methods represent the primary means of controlling this pathogen in warehouses. However, this study sought to explore an alternative approach by harnessing the biocontrol potential of bacterial isolates against brown rot in apple trees. A total of 72 bacterial isolates were successfully obtained from the apple tree rhizosphere and subjected to initial screening via co-cultivation with the pathogen. Notably, eight bacterial isolates demonstrated remarkable efficacy, reducing the mycelial growth of the pathogen from 68.75 to 9.25%. These isolates were subsequently characterized based on phenotypic traits, biochemical properties, and 16S rRNA gene amplification. Furthermore, we investigated these isolates’ production capacity with respect to two enzymes, namely, protease and chitinase, and evaluated their efficacy in disease control. Through phenotypic, biochemical, and 16S rRNA gene-sequencing analyses, the bacterial isolates were identified as *Serratia marcescens*, *Bacillus cereus*, *Bacillus* sp., *Staphylococcus succinus*, and *Pseudomonas baetica*. In dual culture assays incorporating *M. laxa*, *S. marcescens* and *S. succinus* exhibited the most potent degree of mycelial growth inhibition, achieving 68.75 and 9.25% reductions, respectively. All the bacterial isolates displayed significant chitinase and protease activities. Quantitative assessment of chitinase activity revealed the highest levels in strains AP5 and AP13, with values of 1.47 and 1.36 U/mL, respectively. Similarly, AP13 and AP6 exhibited the highest protease activity, with maximal enzyme production levels reaching 1.3 and 1.2 U/mL, respectively. In apple disease control assays, *S. marcescens* and *S. succinus* strains exhibited disease severity values of 12.34% and 61.66% (DS), respectively, highlighting their contrasting efficacy in mitigating disease infecting apple fruits. These findings underscore the immense potential of the selected bacterial strains with regard to serving as biocontrol agents for combatting brown rot disease in apple trees, thus paving the way for sustainable and eco-friendly alternatives to chemical interventions.

## 1. Introduction

Brown rot, which is caused by *Monilinia* spp., is a global scourge, inflicting severe damage on fruit production [1]. Its devastating impact extends to stone fruit trees, including peaches, nectarines, plums, apricots (*M. fructicola*), and cherries (*M. laxa*), as well as seeded fruit trees like apples and pears (*M. fructigena* and *M. laxa*) [2]. Widespread across Australia, Asia, America, and Europe, brown rot wreaks havoc on orchards worldwide [3]. Notably, *M. fructigena* and *M. laxa* have been identified the predominant pathogens responsible for brown rot disease, with a presence observed in major seed and stone fruit production regions, including Iran [4]. Microbial pathogens pose a persistent and significant threat to both food production and ecosystem stability, thereby elevating the challenge of ensuring global food security within a burgeoning population [5,6]. Controlling plant diseases from farm to warehouse is the key to safeguarding food supplies, thus prompting the exploration of physical, chemical, and biological approaches [7]. Yet, the use of chemical controls has generated increasing scrutiny due to potential health hazards, particularly concerning post-harvest plant products in storage facilities. In this context, harnessing the potential of beneficial natural microorganisms presents a viable solution for effective plant disease management [7]. Among the diverse habitats within the microbial world, the rhizosphere stands out as a rich and intricate realm, encompassing plant roots, soil particles, and an array of microorganisms [8,9,10]. Within this complex environment, Plant-Growth-Promoting Rhizobacteria (PGPR) represent a promising group of bacteria sourced from the rhizosphere, offering ecological safety, ensuring food security, and boosting crop productivity [11]. PGPB exhibit the ability to produce a repertoire of metabolites, including antibiotics, siderophores, volatile organic compounds (VOCs), hydrolytic enzymes, and more, effectively reducing or impeding pathogenic diseases [12]. Investigations into rhizosphere microbial diversity across various plant species have unveiled diverse groups of bacteria and fungi, with bacteria emerging as the dominant microorganisms within this niche [13]. These beneficial bacteria play a multifaceted role, stimulating plant growth through phytohormone synthesis, enhancing nutrient availability, inhibiting fungal pathogens, and secreting antibiotics as well as a range of lytic enzymes such as chitinase and protease, thereby promoting sustained plant health and growth [14]. Chitinases, belonging to the glycosyl hydrolase (GH) family, are predominantly produced by bacteria, fungi, insects, plants, and animals. Prominent chitinase-producing bacteria include *Aeromonas*, *Serratia*, *Streptomyces*, and *Bacillus*, with notable examples being *Pseudomonas aeruginosa*, *Bacillus circulans*, and *Serratia marcescens* [15,16,17]. Among the diverse arsenal of hydrolytic enzymes produced by microorganisms, chitinases and proteases have emerged as potent agents for disrupting the cell walls of plant-pathogenic fungi. With their ability to address the environmental risks associated with synthetic fungicide application, chitinolytic enzymes hold promise for agricultural production and the bioremediation of pollutants [18]. The current control measures for *Monilinia* spp. primarily rely on fungicides, which facilitate pathogenic inhibition [19,20]. However, the excessive and continuous use of chemical fungicides can lead to fungicide resistance in *Monilinia* spp. strains and pose various gastrointestinal risks to consumers. Consequently, the quest for determining safe and efficient alternative methods for controlling apple brown rot disease has become imperative. Accordingly, biocontrol bacterial agents, which are capable of producing antifungal compounds such as antibiotics, chitinase enzymes, and proteases, represent compelling candidates for addressing this challenge.

Hence, the primary objective of this scientific study is to isolate and identify biocontrol bacteria with the potential to combat apple brown rot disease in storage conditions, focusing specifically on the production of protease and chitinase enzymes. Through an extensive exploration of the rhizosphere microbiota associated with apple trees, we aim to uncover bacterial isolates that exhibit robust biocontrol properties against this devastating disease. Furthermore, our investigation will emphasize the characterization and quantification of the protease and chitinase enzyme production carried out by these selected bacterial strains, as these enzymes play a crucial role in inhibiting the growth and pathogenicity of the causative agents of apple brown rot disease.

## 2. Materials and Methods

### 2.1. Isolation of Antagonistic Bacteria

For bacterial isolation, soil samples were randomly collected at a depth of 30 cm from different apple orchards in Lorestan Province, Western Iran. Twelve randomly selected clumps of apple tree soil, each consisting of 10–20 plants, were dug up from the field with a fork to a depth of 30 cm (rhizosphere samples) and placed in a large polyethylene bag. At the same time, 12 soil samples (−100 g each) were taken from below the root zone. The samples were stored in plastic bags at 4 °C until the bacteria were isolated. Isolation of bacteria was performed using the dilution series method on a nutrient agar (NA) culture medium at 28 °C. Bacterial colonies were isolated and purified after 48 h [21].

### 2.2. Collection, Isolation, and Identification of Fungal Isolates

*Monilinia laxa*, the causative agent of apple brown rot disease, was isolated from infected apples obtained from orchards in Khorramabad district, Iran. The diseased apple samples were carefully cultured on a potato dextrose agar (PDA) medium and incubated under controlled conditions at 22 °C for 7 days. Subsequently, the fungal isolates were subjected to morphological and morphometrical analyses using the identification key provided by Van Leeuwen et al. [22] to ensure accurate species identification. Furthermore, DNA extraction was performed on the fungal isolates to confirm their identity through molecular studies utilizing specific primers. To maintain the virulence of the pathogenic strain, regular recovery from inoculated apple fruits was conducted, and monoconidial isolates of the fungi were employed for both in vitro and in vivo experiments, thus ensuring the reliability and consistency of the pathogen in subsequent investigations.

### 2.3. Effects of Bacterial Metabolites on the Fungal Pathogen

Bacterial isolates were placed into 100 mL of nutrient broth contained within 300 mL Erlenmeyer flasks. The flasks were then subjected to incubation at 27 °C and 150 rpm for 2 days. Following incubation, the cultures underwent centrifugation (Sigma 3–16 L model, Sigma Laborzentrifugen GmbH, Osterode am Harz, Germany) at 12,000 rpm and 4 °C for 15 min. The resulting supernatant was extracted using 99.8% ethyl acetate at a 1:1 ratio. The upper phase of the extraction was subsequently evaporated in a round-bottom flask under gentle heating at 40 °C, for which a rotary evaporator was employed. A 50 μL aliquot of the extract was carefully applied to a sterile paper disc, which was then allowed to dry. Subsequently, the paper disc, now loaded with the extract, was placed on Potato Dextrose Agar (PDA) plates that were previously inoculated with fungal strains. The plates were subsequently incubated at room temperature (25 °C) for 2 weeks. The presence of a transparent halo around the paper disc, whose diameter was measured in millimeters, was considered indicative of fungal inhibition caused by the extract derived from the bacterial culture [23].

### 2.4. In Vitro Dual Culture Bioassay of Bacterial Isolates versus Monilinia laxa

A preliminary evaluation of the antagonistic effect of the bacterial isolates on *Monilinia* fungus was performed using a dual culture method on a PDA medium. Specifically, 5 mm diameter disks of 7-day-old PDA culture of *M. laxa* were placed in the middle of a petri dish containing PDA. Bacterial strains were inoculated one cm away from the edge of the petri dish containing PDA. The plates were incubated at 25 °C for two weeks. This test was performed with 8 bacterial isolates in three replications (Petri dishes), adopting a completely randomized design. The experiment was repeated twice to reduce possible errors. The antagonistic activity was evaluated by calculating the inhibition rate (IR) of the mycelial growth of *M. laxa* using the following Formula (1):

(1) IR (%)=100×(DC−DT/DC)

where *DC* is equal to the colony diameter in the control treatment and *DT* is equal to the colony diameter in bacterial treatments [23].

### 2.5. In Vivo Antagonism Experiment

For this experiment, uniform and healthy fruits of the Golden delicious cultivar (Golden delicious) were collected from apple orchards in Lorestan Province, Western Iran. Apples were surface-disinfected (specifically, they were immersed in 2% sodium hypochlorite for two minutes, rinsed twice with sterile distilled water, and, finally, immersed in 90% ethyl alcohol for 5 s) and dried on a sterile paper towel. On each apple fruit, one uniform incision (5 mm wide and 3 mm deep) was made at the equator of the apple using a cork borer. Each wound was inoculated with 20 μL of antagonist bacteria at a concentration of 1 × 10^7^ CFU/mL and allowed to dry. The apples were placed in plastic trays containing wet paper towels. The plastic containers were placed into plastic bags and incubated at 15 °C. The treated fruits were then inoculated 24 h afterward with a conidial suspension of the pathogen (20 µL/wound) concentrated at 1 × 10^5^ spores/mL. The fruits were then incubated for 7 and 14 days at 15 °C. The experiment was repeated twice within a given period [24]. The lesion diameters were measured after 7 and 14 days post-inoculation, and the DS of the pathogenic fungus on apples was calculated according to the following formula (2):

(2) DS (%)=(DT)/(DC)×100

where *DT* is the average lesion diameter of the treated wounds, and *DC* is the average lesion diameter of the wounds in the control treatment.

This test was performed with eight antagonistic bacterial isolates, a fungal pathogen, and six apples in each repetition, adopting a completely randomized design. Apple fruits dipped in SDW served as a control. The number of infected fruits was monitored every 7 days until the apples in the control treatments were totally infected. The experiment was repeated three times for greater accuracy and to reduce possible errors.

### 2.6. Gene Sequencing and Phylogenetic Analysis

Isolation of isolated bacteria was performed using phenotypic biochemical tests, including a Gram test [25] and tests of oxidase [26], catalase [27], citrate [28], amylase [29], cellulose [30], and protease production [31], and using 16S rRNA gene sequencing [32]. The 16S rRNA genes of the isolated bacteria were amplified via PCR using the forward primer 27F (5′-AGATTTGATCCTGGCTCAG-3′) and the reverse primer 1492R (5′-GGTTACCTT GTTACGACTT-3′). Materials and amounts used for PCR reaction included 2.5 μL of reaction buffer (PCR buffer 10×), 1.5 μL of MgCl2 (2.5 mM), 0.5 μL of dNTPs (10 mM), 0.25 μL of Taq DNA polymerase (10 U/μL), 1 μL of the 27 F primer (10 mM), 1 μL of the 1492R primer (10 mM), 3 μL of DNA, and 15.25 μL of double distilled water. The program for PCR amplification was performed using a Bio-Rad Thermal Cycler C1000 as follows: initial denaturation at 94 °C (5 min), followed by 30 cycles of denaturation at 94 °C (30 s), annealing at 62 °C (30 s), and extension at 72 °C (2 min), and a final extension at 72 °C (10 min). The PCR products were imaged using gel electrophoresis. Then, 5 μL of PCR products was sent to the 1st BASE (China) for DNA sequencing using an automated DNA sequence. The sequence was compared to the GenBank database available from NCBI using the BLAST search engine to recognize the highest percentage of similarity with the described strains and aligned through the Clustal Multiple Alignment tool. A phylogenetic tree was constructed via the neighbor-joining method using MEGA 6.0 with 1000 bootstraps [33].

### 2.7. Enzyme Assay

#### 2.7.1. Preparation of Colloidal Chitin

Colloidal chitin (CC) was prepared from chitin powder using the method proposed by Alam and Mathur. [34] with minor modifications. Chitin powder (5 gr) was slowly added to 60 mL of concentrated HCl and kept for 24 h at 4 °C. The suspension was mixed with 400 mL of cold ethanol (96%) with vigorous stirring and kept overnight at 4 °C. The precipitate was collected via centrifugation at 12,000 rpm for 10 min and washed several times with distilled water until the colloidal chitin became neutral (pH 7.0). Finally, the supernatant was discarded, and colloidal chitin was used for testing [34]. The final precipitate was dried to a constant weight at 50 °C and stored at room temperature until further use.

The pure bacterial colonies were cultured in liquid NB media supplemented with colloidal chitin 1% (*w*/*v*) and incubated at 40 °C for 48 h on a rotary shaker (180 rpm). After 48 h, the culture nutrient broth (NB) was centrifuged at 12,000 rpm for 10 min at 4 °C, and the supernatant was collected for the measurement of chitinolytic activities [35].

#### 2.7.2. Detection of Chitinolytic Activity on Plates

Chitinolytic activity on plates was detected using the diffusion plate method [36]. Sterilized chitin agar medium containing 1.0 g of colloidal chitin, 2.0 g of agar, and 100 mL of phosphate buffer (8 g/L NaCl, 0.2 g/L KCl, 1.42 g/L Na_2_HPO_4_, and 0.24 g/L KH_2_PO_4_) (pH 7.0) was prepared in Petri dishes [29]. Three wells in each plate were made using a sterile well cutter with a diameter of 7 mm. Forty μL of each supernatant containing chitinase from different strains was added to each well of the plates. The plates were refrigerated at 4 °C for 2 h and then incubated at 28 °C for 24 h. After incubation, the plates were examined to observe and record the clear areas around the wells. The presence of a clear halo around the bacterial wells indicated that chitin hydrolysis had occurred. Each assay was performed three times, and the mean values were calculated. Chitinase activity was determined using the following Formula (3) [36]:

(3) K=D−d

where *K* is the activity of chitinase, *D* is the diameter of the clear region, and *d* is the diameter of the well (*d* = 7 mm).

#### 2.7.3. Determination of Chitinolytic Activity Based on the Colorimetric Method

The chitinolytic activity of each isolate was determined based on the quantities of reducing sugars released from the chitin. A mixture of 1 mL of cell-free supernatant and 1 mL of 1% colloidal chitin suspension was incubated at 40 °C for 1 h. The reaction was completed by adding 1 mL of 1 N NaOH and boiling the mixture at 100 °C for 5 min. After the centrifugation of the reaction mixture at 12,000 rpm for 10 min, 1 mL of a solution containing reducing sugars (N-acetylglucosamine (NAG)) was added to 1 mL of DNS and heated at 100 °C for 10 min. A boiling water bath was prepared. After cooling to room temperature, the solution for OD was measured at ƛ = 535 nm. The rate of reduction of the released sugars was calculated based on the standard NAG curve. The experiment was performed three times, and the results are presented as mean values [37].

#### 2.7.4. Detection of Protease Activity on Plates

Protease activity was assessed by inoculating 20 μL of isolated bacteria on skim milk agar. The bacteria on the agar were then incubated for 24 h at 28 °C. Protease activity was presented at the media level despite a clear area around the colony. Each assay was performed three times, and the mean values were calculated [38].

#### 2.7.5. Determination of Protease Activity Based on the Colorimetric Method

The crude enzyme extract was determined to have been modified by enzyme activity based on a method previously reported by Pratika et al. [39]. A total of 50 μL of the enzymatic solution was added to 50 μL of 0.05 M phosphate buffer at pH 7, and then 50 μL of the substrate (casein 2% in 0.05 M phosphate buffer solution at pH 7) was incubated at 37 °C for 10 min. The reaction was stopped by the addition of 100 μL of 0.4 M horooroacetic acid (TCA). The mix was then centrifuged at 10,000 rpm for 10 min. The supernatant was determined according to its tyrosine colorimetric level. A total of 250 μL of 0.5 M Na_2_CO_3_ was added to each of the 50 μL supernatants and then vortexed for 10 min. Subsequently, 50 μL of Folin–Ciocalteau reagent was added to the mixture, and then the mixture was left to stand for 30 min. Its adsorption solution was read using a UV–Vis nanoparticle spectrophotometer at λ650 nm. Then, the protein concentration in the sample was used to determine protease activity [39].

### 2.8. Statistical Analysis

The data underwent an assessment for normality using the Shapiro–Wilk test. Subsequently, a one-way ANOVA was conducted to assess the variations among the treatments. To determine significant differences, Tukey’s Least Significant Difference (LSD) test was applied at a significance level of 5% (*p* < 0.05) after conducting Levene’s test for variance homogeneity. All datasets were analyzed using SAS software (version 9.4, SAS Institute, Cary, NC, USA).

## 3. Results

### 3.1. Isolation and Screening of Chitinase and Protease-Producing Bacterial Strains

A total of 72 bacterial strains were isolated from 12 soil rhizosphere samples obtained from apple tree areas. Among these, 36 strains were isolated from the Abestan region, accounting for the highest proportion at 50%. The Kamalvand region yielded 14 strains (19.4%), Hossein-Abad yielded 3 strains (4.1%), Domgar contributed 4 strains (5.5%), and Kahriz presented 15 strains (20.9%).

Upon examination, these isolated bacterial strains exhibited distinct morphological characteristics encompassing features such as shape, color, size, gloss, opacity, the regularity or irregularity of margins, length, odor, and texture. The size of these strains ranged from 1 to 3 mm. The colonies formed by the isolates displayed varying attributes such as small, large, or irregularly rounded shapes; raised or flat tops; and colors including white, light green, yellow, or cream. Furthermore, it was observed that the colonies possessed a dry and wrinkled texture.

### 3.2. Identification of Fungal Pathogens

**Figure 1:**
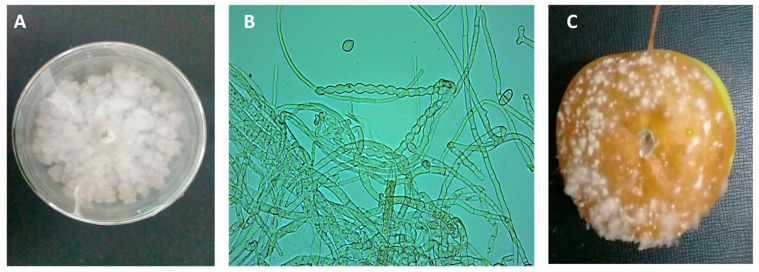
Symptoms in apples caused by *M. laxa*: (**A**) Seven-day-old colony of *M. laxa* on PDA, (**B**) lemon-shaped conidia and mycelium, and (**C**) a brown, rotten fruit covered with white mycelium and spore mass.

*Monilinia laxa* (strain number MLULK30) was isolated from apple blossoms and fruits for the first time in Iran and was recognized as the main cause of blossom burns and brown rot in apple trees. The symptoms of brown rot on the apples in the garden caused by *M. laxa* were accompanied by necrotic areas, gray conidia, and numerous conidiophores on the mycelium. The growth rates of the colony on PDA after 7 days at 22 °C in dark and light conditions were 43.7 and 28.9 mm, respectively. The edge of the colony was rosette-shaped and had gray arches. The conidia were lemon-shaped, and the diameter of the conidia in the culture medium ranged from 9.7 to 13.5 μm (Figure 1). A molecular study based on specific primers showed that all the studied isolates corresponded to *M. laxa*.

### 3.3. Antifungal Activity of Isolated Strains

**Figure 2:**
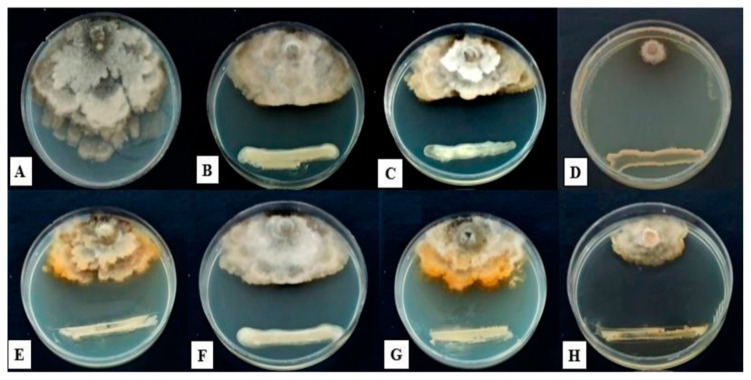
Antagonistic effects of some isolated apple rhizosphere bacterial strains against *M. laxa*: (**A**) control, (**B**) *Pseudomonas baetica*, (**C**) *Pseudomonas baetica*, (**D**) *Serratia marcescens*, (**E**) *Bacillus* sp., (**F**) *Bacillus* sp., (**G**) *Pseudomonas baetica*, and (**H**) *Bacillus cereus*.

**Figure 3:**
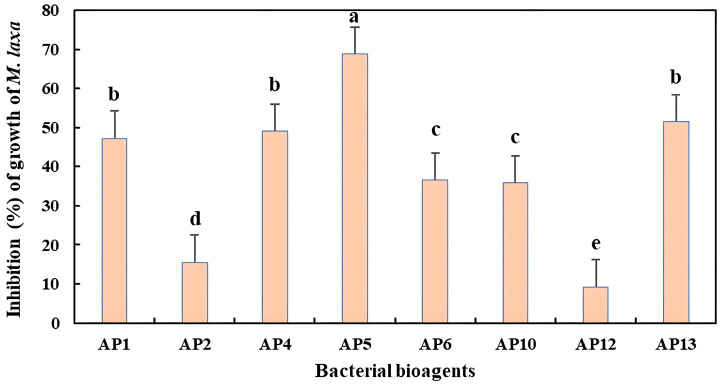
Inhibition rates of mycelial growth of *M. laxa* obtained with volatile organic compounds of bacterial isolates (in dual distance bioassay). Data in the figure represent the means of two independent dependent trials with 3 replicates. In each incubation period, treatments with the same letter (a–e) are not significantly different according to the LSD test (*p* < 0.05).

The results obtained in the investigation of the inhibitory effect of the bacterial isolates on the growth of *M. laxa* mycelia in the dual culture showed significant differences (*p* < 0.05) among the bacterial isolates. Based on the antagonist assay using the direct confrontation method, 8 out of the 72 tested isolates showed good capacity to inhibit fungal growth. The fungal mycelia failed to expand on the agar plates due to bacterial growth. Six isolates showed strong inhibition of *M. laxa* mycelia. Two isolates presented low inhibitory activity against *M. laxa*. All the tested isolates inhibited the mycelial growth of *M. laxa* with various levels of inhibitory activity. The dual culture tests conducted on the bacterial isolates and *M. laxa* revealed that *S. marcescens* and *S. succinus*, presenting values equal to 68.75 and 9.25%, showed the highest and lowest levels of mycelial growth reduction activity, respectively (Figure 2 and Figure 3).

### 3.4. Extraction of Antifungal Compounds

**Figure 4:**
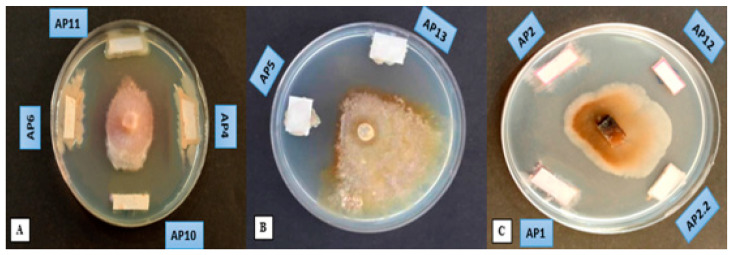
Extraction-of-antifungal-compound-based test assay concerning antagonism of bacterial isolates on *M. laxa*. Different letters indicate different bacterial isolates (**A**–**C**).

Further analysis using bacterial extracts revealed that most of the specimens’ secondary metabolites were unable to inhibit fungal growth, as shown in (Figure 4). Only six isolates, designated as AP1, AP4, AP5, AP6, AP10, and AP13, retained their activity against the fungal strain. The solvent used to extract bioactive compounds may influence the success of the extraction process. Some bioactive compounds may effectively be extracted using a polar solvent and others may not. The concentration of the obtained metabolites also plays an important role in their ability to inhibit fungal growth.

### 3.5. Investigation of the Control Effect of Bacterial Isolates on Apple Brown Rot Disease In Vivo

Two bacterial isolates (*Serratia marcescens* AP5 and *Bacillus cereus* AP13) out of the eight tested bacteria displayed antagonistic activity against *M. laxa* in which 100% inhibition was achieved after 7 days. However, their efficiencies decreased slightly after 14 days of post-incubation as their DS increased from 0 to 0 to 12.34 to 14.71, respectively. However, for the other bacterial isolates, the DS decreased with the increase in the incubation period (Table 1). Statistical analysis of DS underlined that there was a highly significant difference between the bacterial isolates. The results of controlling the bacterial strains on the apples showed that the *S. marcescens* and *S. succinus* strains presenting values of 12.34% and 61.66% had the highest and lowest levels of control of DS on the apples, respectively.

### 3.6. Morphological and Biochemical Characteristics

Eight bacterial isolates with antagonistic properties were identified based on their phenotypic-biochemical characteristics and the sequencing of the 16S rRNA gene. The results regarding the observed phenotypic and biochemical properties are listed in (Table 2).

### 3.7. Phylogenetic Analysis of 16S rRNA Sequences

**Figure 5:**
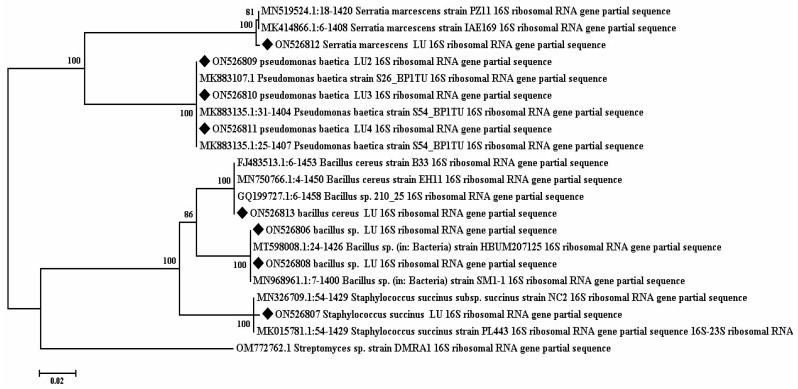
The tree was constructed via the neighbor-joining method using MEGA6 software with 1000 bootstrap replications inferred from the DNA sequences of the 16S rRNA gene. Isolates used in this study and reference isolates were obtained from NCBI GenBank. *Streptomyces* sp. sequence was used to construct the roots of the tree. Nucleotide sequence accession numbers are indicated in parentheses.

In the present study, a total of 72 bacteria were isolated from 12 rhizosphere soil samples collected in an apple tree orchard in Lorestan province, Western Iran. Then, all the bacterial isolates were screened with respect to their ability to inhibit the growth of *M. laxa*; eventually, eight isolates were selected for further analysis. The nucleotide sequences of the 16S rRNA gene (~1500 base pairs) for the strains AP1, AP2, AP4, AP5, AP6, AP10, AP12, and AP13 were determined, and DNA sequencing and analysis using the BLAST tool revealed that the identities of all eight isolates matched available 16srRNA sequences in GenBank at a level ranging from 96% to 100%. Blast analysis of the 16S rRNA sequences in the GenBank database revealed that they generally belong to *P. baetica* (three strains), *S. marcescens* (one strain), *B. cereus* (one strain), *Bacillus* sp. (two strains), and *S. succinus* (one strain) and that the isolated AP1 strains were *P. baetica*, AP2 *P. baetica*, AP4 *P. baetica*, AP5 *S. marcescens,* AP6 *Bacillus* sp., AP10 *Bacillus* sp., AP12 *S. succinus*, and AP13 *B. cereus* (Figure 5).

### 3.8. Results Regarding Protease and Chitinase Activity

**Figure 6:**
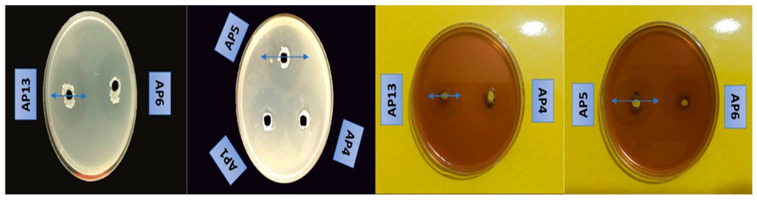
Chitinase and protease activity of a selection of the isolated strains based on their clear zones around the wells after 48 h of incubation.

All isolated strains were screened for chitinase and protease activity based on their clearance region on the colloidal chitin agar and skim milk agar plates as described above. The clearing areas around the wells showed chitinase and protease activity, which can degrade the combination of chitin and protease in the environment. These results show that the rhizosphere soil of apple trees produces great quantities of bacteria with the ability to produce proteases and chitinase. An important feature of these bacteria is their ability to fight plant pathogens. As shown in Table 3 and Figure 6, eight bacterial isolates (AP1, AP2, AP4, AP5, AP6, AP10, AP12, and AP13) were able to produce chitinase and protease. On average, they had zones of 14.36 to 24.16 mm with respect to chitinase production and 15.02 to 25.14 mm in terms of protease production. According to the results obtained via the colorimetric method, out of these eight strains, the six best producers of chitinase and protease and were selected to accurately determine the activity of chitinase and protease via the colorimetric method.

### 3.9. Chitinase and Protease Activity of the Seven Selected Strains Based on the Colorimetric Method

**Figure 7:**
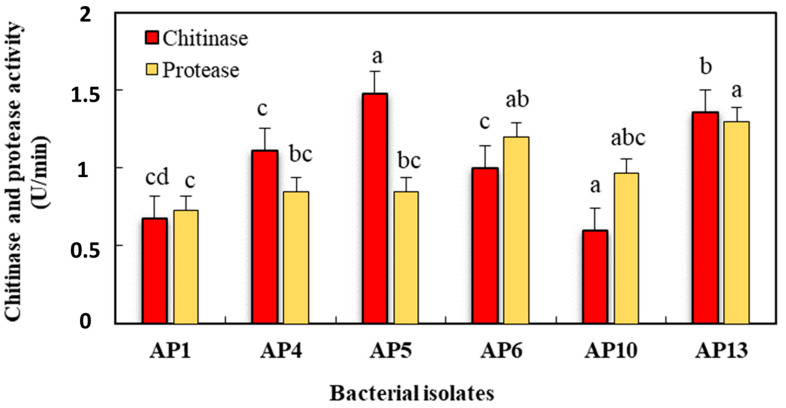
Chitinase and protease activities of the selected bacterial strains are based on measurements of the release of reducing sugars (NAG). For each enzymatic activity, values with the same letters (a–d) are not statistically different according to the LSD test (*p* ≤ 0.05).

The chitinase and protease enzymes secreted by the seven isolated bacterial strains were evaluated using a colorimetric assay, and their activity is illustrated in Table 4 and Figure 7.

Among the six strains, the AP5, AP13, AP4, and AP6 strains yielded the highest levels of chitinase activity, with maximum enzyme production values of 1.47, 1.36, 1.11, and 1 (U/mL) after 48 h of incubation, while AP10 exhibited the lowest chitinolytic activity, namely, 0.6 (U/mL). In the quantitative assay of protease activity, when the protease digests casein, tyrosine is released together with amino acids and other peptide fragments. Among the seven strains, the AP13 and AP6 strains had the highest levels of protease activity, presenting maximum enzyme production levels of 1.3 and 1.2 (U/mL) after 48 h of incubation, while AP1 showed the lowest proteolytic activity, which was equal to 0.7 (U/mL).

## 4. Discussion

Apples (*Malus domestica* Borkh.) hold a prominent position as one of the most popular edible fruits and are of significant economic importance on a global scale [40]. Among the various diseases affecting rosacea family fruits, brown rot caused by *M. laxa* has been widely reported. This disease leads to substantial losses both during storage and in the orchard [41]. In this study, we successfully isolated and identified *M. laxa* from infected fruits based on micromorphological and molecular characteristics, thus confirming this fungus’s pathogenicity. These findings align with the observations made by Holb et al. [42].

Furthermore, eight antagonistic bacterial strains isolated from the rhizosphere of apple trees were evaluated with respect to their capacity to inhibit pathogen growth and produce lytic enzymes of industrial and biotechnological importance under laboratory conditions. The results of our research demonstrated that the majority of the tested bacterial isolates exhibited a significant inhibition rate (over 50%) of *M. laxa* mycelial growth in the dual culture bioassays. These outcomes are consistent with the findings reported by Aiello et al. [43] and Bubici et al. [44], suggesting that these isolates employ diverse mechanisms of action to exert their antagonistic activity, which is tailored to the specific nature of the targeted pathogen. Similar observations were made by Liu et al. [45], who reported the inhibition of the in vitro conidial germination and mycelial growth of *M. fructicola* in the presence of cell-free culture filtrates of *B. amyloliquefaciens* C06, indicating the presence of antifungal compounds. The alteration of *M. laxa*’s mycelial morphology observed in vitro further supports the hypothesis that the antagonistic activity of the isolates is attributed to the direct effects of the inhibitory compounds they release, which have the potential to directly impede pathogen growth or degrade virulence factors and cell wall components [46]. This mode of action may involve the production of antibiotics, oxidase, citrate, and hydrolytic enzymes such as chitinases, proteases, cellulases, and amylases [23,47,48,49]. Hence, bacterial isolates that effectively inhibit mycelial growth in vitro possess the capacity to reduce the infective potential of *M. laxa* in gardens and storage facilities. Accordingly, our isolates, namely, AP5 (*S. marcescens*), AP13 (*B. cereus*), and AP4 (*P. baetica*), represent potential biocontrol candidates, exhibiting the highest antagonistic activity against *M. laxa* mycelial growth. Genera such as *Agrobacterium*, *Arthrobacter*, *Bacillus*, *Cellulomonas*, *Enterobacter*, *Pseudomonas*, and *Serratia* are known for their biocontrol potential against plant diseases [50]. Rachid et al. [51] reported the ability of *B. amyloliquefaciens* B10W10 and *Pseudomonas* sp. B11W11 to reduce brown rot disease severity and incidence in apples. The efficacy of these biocontrol bacteria relies on their capacity to produce lytic enzymes and lipopeptides. Plant-growth-promoting rhizobacteria (PGPRs) represent a diverse group of bacteria isolated from the rhizosphere that are utilized as biological control agents against plant pathogens, offering a promising and reliable alternative to synthetic fungicides [36,52,53]. Lytic enzymes, including chitinases and proteases, play a crucial role in destroying fungal cell walls and impeding fungal growth under laboratory conditions [54]. Bacterial chitinases effectively hydrolyze the cell walls of phytopathogenic fungi by targeting chitin, a major component of the fungal cell wall [7]. Similarly, proteases contribute to the degradation of peptide bonds between amino acids, thus exerting further antifungal effects [55].

Our study involved the statistical optimization of chitinase and protease production, the characterization of enzymatic properties, and the assessment of antifungal activity in order to evaluate the applicatory potential of the isolated strains. The results align with the findings reported by Lahlali et al. [7], highlighting the high levels of chitinase and protease production by certain bacterial isolates. *Bacillus*, *Pseudomonas*, and *Serratia* are well known for their ability to degrade fungal cell walls through the production of lytic enzymes.

In our study, six isolates (AP1, AP4, AP5, AP6, AP10, and AP13) exhibited significant chitinolytic and proteolytic activities, resulting in substantially higher antifungal efficacy compared to other isolated strains. These isolates, therefore, show potential as biocontrol agents against *M. laxa*. The biocontrol activity of rhizobacteria against plant pathogens is not solely attributed to their production of hydrolytic enzymes, such as chitinase and protease, but also to their ability to target chitin-containing structures, which are prime sites for degradation by chitinases. Consequently, phytopathogenic fungi become susceptible to degradation by these enzymes [55,56].

## 5. Conclusions

The escalating concerns associated with the excessive use of toxins in agricultural practices, coupled with the emergence of pathogen resistance, necessitate the exploration of alternative approaches for disease control. In this study, we focused on the identification of antagonist bacteria with biocontrol activity against the causative agent of apple tree brown rot disease, *M. laxa*. The isolation of bacteria from the rhizosphere of apple trees allowed us to select eight bacterial isolates belonging to the genera *Pseudomonas*, *Staphylococcus*, *Bacillus*, and *Serratia* for evaluation.

Among the isolates, AP5, AP13, AP4, and AP1 exhibited significantly higher inhibitory effects on *M. laxa*, displaying a robust suppression of mycelial growth in a potato dextrose agar (PDA) medium. Through dual culture assays conducted on PDA plates, the in vitro antifungal potentials of these four bacterial bioagents against *M. laxa* were quantified as 68.75%, 51.5%, 49%, and 47.25%, respectively, in comparison to the control, after a 7-day incubation period. The control mechanisms employed by these biocontrol bacterial isolates were further elucidated through the secretion of antifungal compounds in the PDA medium along with the production of protease and chitinase enzymes that effectively dismantled the fungal cell wall. Notably, the *Serratia marcescens* strain exhibited a remarkable 79.9% reduction in disease damage, highlighting its potential as a control strategy in combination with other approaches for effectively managing apple brown rot disease in Iran.

By harnessing the biocontrol abilities of these selected bacterial isolates, the risks associated with the excessive use of toxins can be effectively mitigated, thereby ensuring the safety of consumers while simultaneously countering the development of pathogen resistance. The isolation of these bacteria from the apple tree rhizosphere underscores their natural prevalence and suitability for implementation in sustainable disease management strategies. This study not only contributes to the expanding body of knowledge regarding biocontrol agents but also presents a novel and promising avenue for combating apple brown rot disease.

## Figures and Tables

**Table 1:** Disease severity (%) of brown rot disease (*Monilinia laxa*) as influenced by bacterial isolates and pathogen pressure 7 and 14 days post-incubation at 15 °C.

Strain No.	BLAST Research Results	Accession	Disease Severity (%) 7 Days	Disease Severity (%) 14 Days
AP1	*Pseudomonas baetica*	ON526810	44.31 ^d^	31.18 ^e^
AP2	*Pseudomonas baetica*	ON526809	49.03 ^e^	35.89 ^f^
AP4	*Pseudomonas baetica*	ON526811	24.46 ^b^	24.66 ^c^
AP5	*Serratia marcescens*	ON526812	00.00 ^a^	12.34 ^a^
AP6	*Bacillus* sp.	ON526806	34.25 ^c^	27.98 ^d^
AP10	*Bacillus* sp.	ON526808	52.11 ^f^	37.90 ^g^
AP12	*Staphylococcus succinus*	ON526807	89.33 ^h^	61.66 ^i^
AP13	*Bacillus cereus*	ON526813	00.00 ^a^	14.71 ^b^

Identities of antifungal rhizosphere bacteria based on 16S rRNA gene analysis. In the columns, values with the same letter are not statistically different according to the LSD test (*p* ≤ 0.05).

**Table 2:** Morphological and biochemical tests conducted to identify the strains.

No.	Strain	Gram	Oxidase	Catalase	Citrate	Amylase	Cellulase	Protease
**1**	AP1	−	+	+	+	+	−	+
**2**	AP2	−	+	+	+	−	−	+
**3**	AP4	−	+	+	+	+	+	+
**4**	AP5	−	−	+	+	+	+	+
**5**	AP6	+	+	+	+	+	+	+
**6**	AP10	+	−	+	+	+	−	+
**7**	AP12	+	−	+	+	+	+	+
**8**	AP13	+	−	+	+	+	+	+

(+): positive reaction; (−): negative reaction.

**Table 3:** Screening of bacterial isolates for chitinase and protease activity.

BLAST Research Results	Bacterial Isolates	Chitinase Activity (k, mm)	Protease Activity (k, mm)
*Pseudomonas baetica*	AP1	20.11	22.31
*Pseudomonas baetica*	AP2	14.63	16.54
*Pseudomonas baetica*	AP4	21.02	23.11
*Serratia marcescens*	AP5	24.16	25.14
*Bacillus* sp.	AP6	20.13	21.68
*Bacillus* sp.	AP10	20.05	20.89
*Staphylococcus succinus*	AP12	14.36	15.02
*Bacillus cereus*	AP13	23.54	24.87

Notes: k < 15 mm: weak protease and chitinase activity; 15 mm ≤ k < 20 mm: medium protease and chitinase activity; 20 mm ≤ k < 25 mm: strong protease and chitinase activity; k ≥ 25 mm: very strong protease and chitinase activity.

**Table 4:** Capacity of bacterial antagonists to produce protease and chitinase enzymes involved in biocontrol mechanism production.

Strain No.	Chitinase Activity (U/min)	Protease Activity (U/min)
AP1	0.675 ^cd^	0.725 ^c^
AP4	1.11 ^c^	0.85 ^bc^
AP5	1.475 ^a^	0.85 ^bc^
AP6	1 ^c^	1.2 ^ab^
AP10	0.6 ^d^	0.975 ^abc^
AP13	1.36 ^b^	1.3 ^a^

Data are the averages of three replications ± the standard error of the means. In the columns, values with the same letter (a–d) are not statistically different according to the LSD test (*p* ≤ 0.05).

## Data Availability

The data presented in this study are available on request from the corresponding author.
